# Supplementary material for: Development of type 2 diabetes and insulin resistance in people with HIV infection: Prevalence, incidence and associated factors
Source: PLoS One. 2021 Jun 30;16(6):e0254079. doi: 10.1371/journal.pone.0254079 (PMC8244855; doi:10.1371/journal.pone.0254079)
Supplement: Attachment — Submitted filename: PLOSONE_Review_DM prevalence in HIV_2021.docx [file pone.0254079.s002.docx]

The manuscript by Bratt et al. demonstrates a nice prospective study to evaluate the incidence of type 2 diabetes (T2D) and insulin resistance among a cohort of 570 people living with HIV. While the topic is not novel, this study provides additive data to ongoing literature describing prevalence and incidence of T2D in people living with HIV in different countries. The following are suggested changes to add clarity and rigor to the manuscript.

Major Changes:

- The ‘*total study population’* is described as a cohort of 570 patients including those with T2D and T1D at baseline and the ‘*prospective study population’* as a cohort of 505 patients excluding those with T2D and T1D at baseline.
  - It is unclear to me why the authors compare these 2 cohorts when the primary outcome is incidence of T2D. I recommend the authors either better justify why they are including those with baseline diabetes (both T1D and T2D) in the analyses or exclude the ‘*total study population’* in the analyses.

Minor Changes:

- **Methods** section: Please include the inclusion/exclusion criteria
  - Justify why you only include those >50 years old.
- **Procedure and follow up** section: Please be explicit in how many ‘repeated’ measures of fasting glucose, cholesterol, BP measurements, etc were needed or done before diagnosing participants with T2D, hyperlipidemia, HTN, etc.
- Page 7, ‘**Description of the population data at baseline’** section: Paragraph that starts with “more than 95% of the patients were on cART…. The second sentence uses the word “respectively”- but it is unclear which groups you are ‘respectively’ referring to. Please clarify.
- ‘**Prevalence of T1D, T2D and insulin resistance at baseline and follow up’** section: 2^nd^ sentence needs clarification- what follow up are you referring to when describing the 10% of T2D diagnosed- the 58 patients with T2D were those with T2D at baseline, correct?
- Same section: sentence that begins: “During the follow up 69% (40/58) of the patients diagnosed with T2D…” – the numbers you report do not add up to 100%. I believe the “21%” should be 31%- please check the math.
- Same section: Last sentence of this section that begins with “Death occurs in 8%...”- needs grammatical attention.
- **Discussion** section: Need more detail on the association of T2D and HLA B5701- compare with other literature.
- Generally: I advise the authors work with a writing coach or copyeditor to improve the flow and readability of the text. There are minor grammatical edits that should be addressed throughout the paper.
