# Supplementary material for: Development of type 2 diabetes and insulin resistance in people with HIV infection: Prevalence, incidence and associated factors
Source: PLoS One. 2021 Jun 30;16(6):e0254079. doi: 10.1371/journal.pone.0254079 (PMC8244855; doi:10.1371/journal.pone.0254079)
Supplement: Attachment — Submitted filename: Responses to the reviewers.docx [file pone.0254079.s003.docx]

**Responses to the reviewers’ specific comments:**

**Reviewer # 1:**

**Response:** We thank this reviewer for constructive comments on the manuscript.

Re: Introduction

1. “The inclusion of HLA B 5701 as part of the 4th paragraph seems random. There is not enough information provided as to its relevance and expanding on why this marker was chosen would provide clarity, perhaps as a paragraph of its own. This is especially relevant since HLA B 5701 is a focus of the results and discussion”

**Response**: Thanks for this important comment. We have added more information about, and why, HLA B 5701 was included in the study. To our knowledge this has not been previously examined. We have now inserted following text in the revised manuscript (Introduction): *“The HLA B 5701 allele was recently suggested to protect against type 1 diabetes (T1D) in the large international Type 1 Diabetes Genetics Consortium (T1DGC) study [19]. This allele is also the only genetic marker routinely screened for in HIV-care in order to avoid abacavir hypersensitivity [20] . In addition to indicating abacavir hypersensitivity the HLA B 5701 allele is also associated with restriction of HIV-replication in long-term non-progressors [21]. Whether HLA B 5701 expression also has an impact on T2D development has, to the best of our knowledge, not been investigated.”*

Re: Material and Methods

1.”Remove the additional words 'at baseline' in this title since the longitudinal aspect of the study is described as well”

**Response**: The words 'at baseline' has been removed and the title is now *“Material and Methods”*

2.”Explain why the ref 20 (Koppel K et al) as part of the 4^th^ paragraph seems random”.

**Response**: Reference 20 (Koppel et al.) has been moved to a different place in the text (Procedure and follow up): *“Since the introduction of protease-inhibitor based cART (PI-cART) in 1996 there has been a special interest in metabolic and hemostatic parameters in our clinic [20] and a yearly evaluation of all patients includes the fasting testing…”*

3. “Provide evidence that a fasting HOMA-IR >=3.0 is diagnostic of insulin resistance in PLHIV”.

**Response**: This point is well taken. We have included more text motivating this cut off level and inserted two more references (24, 25) to justify cut off value of 3 and above. Also, our own results and clinical experience from cross-sectional HOMA examinations in 455 50+, HIV+ patients were contributed to this value. Following text has been inserted in the revised manuscript (Procedure and follow up): *“For the definition of insulin resistance in non-diabetic patients HOMA-IR ≥3.0 was used (24). This value was close to the median of 2.7 (interquartile range: 1,8-4,1) among non-diabetic HIV-patients 50 years or older in our clinic in 2020 (n=455), and similar to the cut-off value defining insulin resistance in the BRAMS study [25]”*

4. “In the last sentence of this section, it states that a BMI≥30 kg/m2 was used to define central adiposity. BMI is not a measure of central adiposity”. **Response**: We thank for this comment. BMI≥30 kg/m2 has been omitted as a measure of central adiposity (Procedure and follow up): *“Central obesity was defined by waist circumference ≥94 cm and ≥80 cm for men and women, respectively”.*

5. “The term Total Clinic Population is used along with Total Study Population to describe the same group. Only one title should be presented”.

**Response:** Many thanks for this comment. In the revised manuscript the term Total Clinic Population has been omitted

Re: Results:

1. “It would be my suggestion to remove the data presented in the results section on the Total Study Population since the focus of the article is on the Prospective Cohort. Inclusion of the data on the total group makes the results more confusing without adding to the findings. Stating that the initial Total Study Population presented with n=7 with T1D and n=58 with T2D is the only information that is needed. All other data presented should be only on the Prospective Cohort, pre and post follow-up.”

**Response**: Again, many thanks for helping us in a more straightforward way show our data. We have, in the revised manuscript, removed the data on *Total Study Population* from *Table 1* and now only include the data for the *Prospectively Followed Cohort*. The paragraph has been changed to read (Patients [Material and Methods]): *“At baseline the 570 eligible patients were analysed cross-sectionally. Of these, one percent (7/570) had T1D and 10% (58/570) had T2D. Of the T2D patients 69% (40/58) were treated with glucose lowering medication: 14 individuals were on insulin only, 20 on metformin only and 6 on insulin in combination with metformin; 31% (18/58) were treated with diet and physical activation only. The remaining 505 patients without diabetes (referred to as “the cohort”) were prospectively followed with yearly routine testing in the clinic for the development of the outcomes of interest, i.e. T2D and insulin resistance”.*

**Table 1.** Descriptive population data of the patients without diabetes (n=505) at baseline

|  | **Patients** | |
| --- | --- | --- |
| **N (M; F, n (%))** | | **505 (M 440 (88%); F 65 (12%))** |
| **Age (median (range); years** | | **57 (49-83)** |
| **HLA B 5701 pos (n; (%))** | | **21/500 (4%)** |
| **Time with known HIV-infection**  **(months; median (range))** | | **204 (6-374)** |
| **AIDS diagnosis (n; (%))** | | **94 (19%)** |
| **Initial, pre-ART, HIV-RNA**  **(median (range); copies/ml)** | | **42100 (19-10000000)** |
| **CD4 nadir (x10^6^/L; median; (range))** | | **193 (0-870)** |
| **CD4 nadir <200 x10^6^/L (n; (%))** | | **266 (53%)** |
| **Time without cART (median (range); months)** | | **54 (0-372)** |
| **Treatment interruption of at least one month: prevalence (%); time (median (range); months)** | | **26%; 16.0 (1-105)** |
| **Ever on D4t (Stavudine) (n; (%))** | | **179 (35%)** |
| **Ever on DDI (Didanosine) (n; (%))** | | **179 (35%)** |
| **On cART (n; (%))** | | **491 (97%)** |
| **cART including a NNRTI (n; (%))** | | **306 (61%)** |
| **cART including a Protease inhibitor (n; (%))** | | **162 (32%)** |
| **cART including an Integrase inhibitor (n; (%))** | | **100 (20%)** |
| **cART including Emtricitabine (n; (%))** | | **260 (51%)** |
| **cART including Lamivudine (n; (%))** | | **193 (38%)** |
| **cART including Abacavir (n; (%))** | | **176 (35%)** |
| **cART including Tenofovir (n; (%))** | | **268 (53%)** |
| **cART including Zidovudine (n; (%))** | | **5 (1%)** |
| **CD4 count (median; (range))** | | **600 (30–1620)** |
| **HIV-RNA <100 copies/ml at baseline (n; (%))** | | **489 (97%)** |
| **Hypertension (n; (%))** | | **205 (41%)** |
| **Hyperlipidemia (n; (%))** | | **202 (40%)** |
| **Central obesity** | | **114 (23%)** |
| **Insulin resistance (n; (%))** | | **153 (30%)** |
| **On statin treatment (n; (%))** | | **115 (23%)** |

2. “It states in methods that there were n=570 in the Total Study Population and that the 505 individuals without a diagnosis of diabetes were prospectively followed. If that is the case, please clarify the central adiposity numbers presented in table 1. There is only an n=65 difference between the two groups in total numbers”.

**Response**: Table 1 now only contains data on the Prospectively followed cohort. The incorrect figures for central obesity have been corrected (Table 1 above)

3. “The relevance of table 4 isn't clear. There needs to be more development of the importance of HLA B 5701 in the introduction”.

**Response**: Table 4 has been removed. In the introduction we have included further motivation of why we included HLA B5701 in the study: See response #1

We have also inserted new text (which replace Table 4) in the revised manuscript for the association between HLA B5701 at baseline and at follow up (Characteristics of the HLA B5701 positive patients [Results]: *“At baseline, regarding all patients (n=570), T2D was more common among HLA B 5701 positive as compared to HLA B5701 negative patients (22% (6/27) vs 10% (52/531); p=0.05). Expressing HLA B5701 and having been HIV-positive longer in months than the median (HIV-months >209 months) were significantly related to the presence of T2D in univariate analysis. In multivariate analysis, controlled for age, only being HLA B 5701 positive showed a trend (p=0.055) for association with T2D, Table 2.* *However, during follow up there was no difference in new T2D diagnoses between HLA B 5701 positive and negative patients (14% (3/21) vs 8% (39/479); ns). None of the patients with T1D were HLA B 5701 positive (0/7).”*

Re: Discussion:

1. “The findings suggest that predictive factors underlying the development of T2D in PLHIV are similar to the general population and not related to the HIV infection itself. There needs to be a paragraph added to the discussion regarding this finding since there are a number of other studies suggesting that HIV-related factors do contribute to the increased prevalence of T2D in PLHIV.”

**Response**: Thanks for this point. We have added, in the revised manuscript, a paragraph and two more references from 2018 (Nausseau JR and Echecopai-Sabogal J) regarding HIV-related factors that contribute to the increased prevalence of T2D in PLHIV found in other studies (Discussion): “*A systematic review and meta-analysis in 2008 found an association between PIs and the metabolic syndrome but not T2D [40]. However, the metabolic syndrome or combinations of its components might develop into T2D as our results indicate. In a more recent metanalysis from 2018 the major risk factors, apart from aging, for diabetes and prediabetes were found to be family history of diabetes, Black or Hispanic origin, overweight/obesity, central obesity, lipodystrophy/lipoatrophy, dyslipidemia, metabolic syndrome, increased baseline fasting glycemia, and certain cART regimens [34]. Integrase inhibitors, mainly Dolutegravir, have been anectodically associated to T2D, possibly due to weight gain, which we could not confirm. We failed to find any influence on T2D of different cART regimes or of treatment interruption. The only integrase inhibitor used in our clinic in 2012 was Raltegravir.*

The manuscript has thoroughly been revised regarding spelling and grammatical errors by a native English speaker.

**Reviewer #2:**

We would like to thank this reviewer for valuable critics which has strengthen the manuscript.

Re: Major Changes:
- The ‘total study population’ is described as a cohort of 570 patients including those with T2D and T1D at baseline and the ‘prospective study population’ as a cohort of 505 patients excluding those with T2D and T1D at baseline.
o It is unclear to me why the authors compare these 2 cohorts when the primary outcome is incidence of T2D. I recommend the authors either better justify why they are including those with baseline diabetes (both T1D and T2D) in the analyses or exclude the ‘total study population’ in the analyses.

**Response**: This point is well taken (which also was raised by reviewer #1 issue #5). We thank for the advice to rearrange our cohort. We have, in the revised manuscript, removed the data on *Total Study Population* from *Table 1* and now only include the data for the *Prospectively Followed Cohort*. The paragraph has been changed to read (Patients [Material and Methods]): *“At baseline the 570 eligible patients were analysed cross-sectionally. Of these, one percent (7/570) had T1D and 10% (58/570) had T2D. Of the T2D patients 69% (40/58) were treated with glucose lowering medication: 14 individuals were on insulin only, 20 on metformin only and 6 on insulin in combination with metformin; 31% (18/58) were treated with diet and physical activation only. The remaining 505 patients without diabetes (referred to as “the cohort”) were prospectively followed with yearly routine testing in the clinic for the development of the outcomes of interest, i.e. T2D and insulin resistance”.*

**Table 1.** Descriptive population data of the patients without diabetes (n=505) at baseline

|  | **Patients** | |
| --- | --- | --- |
| **N (M; F, n (%))** | | **505 (M 440 (88%); F 65 (12%))** |
| **Age (median (range); years** | | **57 (49-83)** |
| **HLA B 5701 pos (n; (%))** | | **21/500 (4%)** |
| **Time with known HIV-infection**  **(months; median (range))** | | **204 (6-374)** |
| **AIDS diagnosis (n; (%))** | | **94 (19%)** |
| **Initial, pre-ART, HIV-RNA**  **(median (range); copies/ml)** | | **42100 (19-10000000)** |
| **CD4 nadir (x10^6^/L; median; (range))** | | **193 (0-870)** |
| **CD4 nadir <200 x10^6^/L (n; (%))** | | **266 (53%)** |
| **Time without cART (median (range); months)** | | **54 (0-372)** |
| **Treatment interruption of at least one month: prevalence (%); time (median (range); months)** | | **26%; 16.0 (1-105)** |
| **Ever on D4t (Stavudine) (n; (%))** | | **179 (35%)** |
| **Ever on DDI (Didanosine) (n; (%))** | | **179 (35%)** |
| **On cART (n; (%))** | | **491 (97%)** |
| **cART including a NNRTI (n; (%))** | | **306 (61%)** |
| **cART including a Protease inhibitor (n; (%))** | | **162 (32%)** |
| **cART including an Integrase inhibitor (n; (%))** | | **100 (20%)** |
| **cART including Emtricitabine (n; (%))** | | **260 (51%)** |
| **cART including Lamivudine (n; (%))** | | **193 (38%)** |
| **cART including Abacavir (n; (%))** | | **176 (35%)** |
| **cART including Tenofovir (n; (%))** | | **268 (53%)** |
| **cART including Zidovudine (n; (%))** | | **5 (1%)** |
| **CD4 count (median; (range))** | | **600 (30–1620)** |
| **HIV-RNA <100 copies/ml at baseline (n; (%))** | | **489 (97%)** |
| **Hypertension (n; (%))** | | **205 (41%)** |
| **Hyperlipidemia (n; (%))** | | **202 (40%)** |
| **Central obesity** | | **114 (23%)** |
| **Insulin resistance (n; (%))** | | **153 (30%)** |
| **On statin treatment (n; (%))** | | **115 (23%)** |

Re: Minor Changes

Re: Methods section:

Please include the inclusion/exclusion criteria

**Response**: We have now included inclusion and exclusion criteria in the revised manuscript (Patients [Material and Methods]:*“Inclusion criteria for the study were: 1. Having a verified HIV-1 infection being treated at Venhälsan, South Hospital, Stockholm. 2. Born 2012 or earlier. 3. Had given verbal consent to participate.*

*Exclusion criteria were: Having been diagnosed with T1D or T2D prior to the start of the study and refusing to participate”*

Justify why you only include those >50 years old.

**Response**: We have now in the revised manuscript explained the reason for only including >50 years old patients (Introduction): “*The aim of this study was to investigate the incidence of T2D and insulin resistance in a group of well controlled PLHIV over 50 years of age and over a long-term period. The decision to only include this age group was based on our clinic´s focus on co-morbidity in an aging HIV-population.”*

Re: Procedure and follow up section:

Please be explicit in how many ‘repeated measurements of fasting glucose, cholesterol, BP etc were needed or done before diagnosing participants with T2D, hyperlipidemia, HTN, etc.

**Response**: This point is well taken. We have in the revised manuscript now clarified how many ‘repeat measurements of fasting glucose, cholesterol and BP measurements that were carried out before diagnosing participants with T2D, hyperlipidemia and hypertension (Procedure and follow up): *”T2D was defined as repeated fasting glucose values ≥7.0 mmol/l [26]. Hyperlipidaemia was defined as repeated values of either total cholesterol ≥5.0 mmol/l, LDL cholesterol ≥3.0 mmol/l or on-going lipid lowering therapy. Pathological fasting glucose and lipid values were verified at least once. Hypertension was defined by repeated BP ≥140/90 mmHg on at least two different occasions or on-going antihypertensive treatment”.*

- Page 7, ‘Description of the population data at baseline’ section: Paragraph that starts with “more than 95% of the patients were on cART…. The second sentence uses the word “respectively”- but it is unclear which groups you are ‘respectively’ referring to. Please clarify.

**Response**: Thanks for this comment. The word “respectively”- has been excluded and the sentence rewritten (Description of the prospectively followed cohort): *“More than 95% of the patients were on cART and over 95% had HIV-RNA <100 copies/ml. A previous history of advanced immune deficiency and AIDS had occurred in 50% and 20%, respectively, Table 1. Furthermore, 29% had had a treatment interruption of at least one month. The median time of treatment interruption was 14.5 months (range: 1-105 months). The CD4 count had increased from less than 200 x10^6^/L to 600 x10^6^/L, Table 1”.*

- The prevalence of T1D, T2D and insulin resistance at baseline and follow up’ section: 2nd sentence needs clarification- what follow up are you referring to when describing the 10% of T2D diagnosed- the 58 patients with T2D were those with T2D at baseline, correct? ‘

**Response**: Thanks for this comment. It is correct that the 58 patients with T2D are those with T2D at baseline. However, this has now been clarified that this 570 was analysed cross-sectional due to HLA B5701 and excluded in the prospectively followed cohort. Following text has been inserted to further clarify this (Patients [Materials and Methods]):*“At baseline the 570 eligible patients were analysed cross-sectionally. Of these, one percent (7/570) had T1D and 10% (58/570) had T2D. Of the T2D patients 69% (40/58) were treated with glucose lowering medication: 14 individuals were on insulin only, 20 on metformin only and 6 on insulin in combination with metformin; 31% (18/58) were treated with diet and physical activation only. The remaining 505 patients without diabetes (referred to as “the cohort”) were prospectively followed with yearly routine testing in the clinic for the development of the outcomes of interest, i.e. T2D and insulin resistance.”*

- Same section: sentence that begins: “During the follow up 69% (40/58) of the patients diagnosed with T2D…” – the numbers you report do not add up to 100%. I believe the “21%” should be 31%- please check the math.

**Response**: We have checked the figures and “21%” should be “31%”. This has now been corrected: “*Of the T2D patients 69% (40/58) were treated with glucose lowering medication: 14 individuals were on insulin only, 20 on metformin only and 6 on insulin in combination with metformin; 31% (18/58) were treated with diet and physical activation only”.*

- Same section: Last sentence of this section that begins with “Death occurs in 8%...”- needs grammatical attention.

**Response**: The sentence has been grammatically corrected to:” *Death occurred in 8% (42/505) (mortality rate 1.2/100 patient-years)”.*

Re: Discussion section

Need more detail on the association of T2D and HLA B5701- compare with other literature.

**Response**: This point is well taken. We now discuss the association of T2D and HLA B5701 in more depth. We have, however, failed to find any references on B 5701 in relation to T2D. So, there seems to be no studies of the relation between HLA B5701 and T2D in HIV. Following text has been inserted in the revised manuscript (Discussion):*“The trend suggesting a possible association of T2D with HLA B5701 at baseline was surprising and difficult to explain as HLA B5701 has been found to be protective for T1D [19]. One explanation might be a survival bias since time with HIV-infection was also associated to T2D in univariate regression analyse at baseline and HLA B5701 is associated with restriction of HIV-replication in long-term non-progressors [21]. The HLA B5701 link needs further studying.”*

The manuscript also been revised regarding spelling and grammatical errors by an English speaker.
