# Supplementary material for: Development of type 2 diabetes and insulin resistance in people with HIV infection: Prevalence, incidence and associated factors
Source: PLoS One. 2021 Jun 30;16(6):e0254079. doi: 10.1371/journal.pone.0254079 (PMC8244855; doi:10.1371/journal.pone.0254079)
Supplement: Attachment — Submitted filename: Response to the reviewers_R2.docx [file pone.0254079.s004.docx]

**Responses to the reviewers’ specific comments:**

**Reviewer # 1:**

Re: Minor concerns

The remaining grammatical errors including missing periods have been corrected. The use of comms and decimal point has been made consistent throughout the manuscript and in Tables II and III.

Re: Introduction

First sentence: “Mortality and morbidity among people living with human immunodeficiency virus type 1 infection has decreased over the last two decades” has been changed to *“Mortality and morbidity among people living with human immunodeficiency virus type 1 infection have decreased over the last two decades”*

Re: Material and Methods

Patients: Inclusion criteria number 2 needs to be corrected

**Response**: Inclusion criteria number 2 has been clarified to: *“Born before the 1^st^ of January 2013”*

Re: Results:

1 Development of T2D and insulin resistance at follow up – Second sentence reads “Another 7% (35/505) (M:n=19; F:n=17) developed insulin resistance (incidence of 1.1/100 patient-years)”. However, table 1 indicates that 135/505 of the prospectively followed cohort already had insulin resistance at baseline. This would indicate that 36 of the remaining 352 individuals developed insulin resistance. Please clarify here and in discussion.

**Response**: We have made new calculations and rewritten the sentence to: “*During a median follow-up time of 7.25 years (3485 patient-years) 9% (43/505) (M: n=43; F: n=0) developed T2D (incidence of 1.2/100 patient-years). Another 36 patients (M: n=19; F: n=17) developed insulin resistance (incidence of 1.0/100 patient-years). Thus, at follow up, 79 patients (M: n=62; F: n=17) had developed either T2D or insulin resistance (incidence of 2.3/100 patient-years). In total, the occurrence of either T2D or insulin resistance was 46% (232 (153+79)/505) at follow up, Figure 2”.*

We also added the sentence: *”Insulin resistance occurred in 30% (153/505), Table 1”* in the first part of the Results: **Description of the Prospectively Followed Cohort** for further clarification.

The Table 1 and Tables 2-3 have been slightly reorganized for clarification and improved symmetry as shown:

**Table 1.** Descriptive population data of the patients without diabetes (n=505) at baseline.

|  | Patients | |
| --- | --- | --- |
| Total number (M; F, n (%)) | | **505 (M 440 (88%); F 65 (12%))** |
| Median age in years (range) | | **57 (49-83)** |
| HLA B 5701 pos n; (%) | | **21/500 (4%)** |
| Median time with known HIV-infection  in months (range) | | **204 (6-374)** |
| AIDS diagnosis n; (%) | | **94 (19%)** |
| Median initial, pre-ART, HIV-RNA  in copies/ml (range) | | **42100 (19-10000000)** |
| Median CD4 nadir x10^6^/L median (range) | | **193 (0-870)** |
| CD4 nadir <200 x10^6^/L n; (%) | | **266 (53%)** |
| Median time in months without cART (range) | | **54 (0-372)** |
| Treatment interruption of at least one month: prevalence in %; median time in months (range) | | **26%; 16.0 (1-105)** |
| Ever on D4t (Stavudine) n; (%) | | **179 (35%)** |
| Ever on DDI (Didanosine) n; (%) | | **179 (35%)** |
| On cART n; (%) | | **491 (97%)** |
| cART including a NNRTI n; (%) | | **306 (61%)** |
| cART including a Protease inhibitor n; (%) | | **162 (32%)** |
| cART including an Integrase inhibitor n; (%) | | **100 (20%)** |
| cART including Emtricitabine n; (%) | | **260 (51%)** |
| cART including Lamivudine n; (%) | | **193 (38%)** |
| cART including Abacavir n; (%) | | **176 (35%)** |
| cART including Tenofovir n; (%) | | **268 (53%)** |
| cART including Zidovudine n; (%) | | **5 (1%)** |
| Median CD4 count x10^6^/L (range) | | **600 (30–1620)** |
| HIV-RNA <100 copies/ml at baseline n; (%) | | **489 (97%)** |
| Hypertension n; (%) | | **205 (41%)** |
| Hyperlipidemia n; (%) | | **202 (40%)** |
| Central obesity n; (%) | | **114 (23%)** |
| Insulin resistance n; (%) | | **153 (30%)** |
| On statin treatment n; (%) | | **115 (23%)** |

**Table 2.** Uni and multivariate analysis regarding T2D in all patients including patients with diabetes (n=570) at baseline. The multivariate analysis was controlled for age. All parameters with p<0.20 in the univariate analysis were entered in the multivariate equation. OR and 95% confidence interval.

|  | Univariate  OR (95% CI) | P-value | Multivariate  OR (95% CI) | P-value |
| --- | --- | --- | --- | --- |
| HLA B 5701 positive | **2.6 (1.0-6.8)** | 0.046 | **2.6 (1.0-7.1)** | 0.055 |
| HIV-months >the median (209 months) | **1.9 (1.1-3.3)** | 0.03 |  | ns |
| Ever an AIDS diagnosis | **1.7 (1.0-3.1)** | 0.11 |  | ns |
| First HIV-RNA >300000 copies/ml | **1.4 (0.8-2.5)** | 0.21 |  |  |
| CD4 nadir <200 x 10^6^/L | **1.7 (1.0-3.0)** | 0.065 |  | ns |
| CD4<200 x 10^6^/L for >12 months | **1.7 (1.0-3.1)** | 0.061 |  | ns |
| Ever on D4t (Stavudine) | **1.4 (0.8-2.4)** | 0.25 |  |  |
| Ever on DDI (Didanosine) | **1.7 (1.0-2.9)** | 0.057 |  | ns |
| Ever been a smoker | **1.4 (0.8-2.4)** | 0.29 |  |  |

**Table 3.** HIV-parameters, treatment, metabolic and inflammatory factors at baseline in relation to T2D development during follow up (n=505)*.* Uni and multivariate analysis at baseline. The multivariate analysis was controlled for age. All parameters with p<0.20 in the univariate analysis were entered in the multivariate equation. Odds Ratio (OR) and 95% confidence interval.

|  | Univariate  OR (95% CI) | P-value | Multivariate  OR (95% CI) | P-value |
| --- | --- | --- | --- | --- |
| HLA B 5701 positive | **1.6 (0.5-5.5)** | 0.46 |  |  |
| HIV-months >the median (209 months) | **0.8 (0.4-1.5)** | 0.52 |  |  |
| Ever an AIDS diagnosis | **0.8 (0.3-1.8)** | 0.59 |  |  |
| First HIV-RNA >300000 copies/ml | **0.8 (0.4-1.6)** | 0.59 |  |  |
| CD4 nadir <200 x 10^6^/L | **1.4 (0.7-2.7)** | 0.31 |  |  |
| CD4<200 x 10^6^/L for >12 months | **0.9 (0.4-1.9)** | 0.76 |  |  |
| Ever on D4t (Stavudine) | **0.9 (0.5-1.7)** | 0.88 |  |  |
| Ever on DDI (Didanosine) | **1.2 (0.6-2.3)** | 0.60 |  |  |
| On an NNRTI | **0.9 (0.5-1.6)** | 0.68 |  |  |
| On a protease inhibitor | **1.2 (0.6-2.2)** | 0.68 |  |  |
| On an integrase inhibitor | **0.6 (0.2-1.4)** | 0.22 |  |  |
| On abacavir | **1.5 (0.8-2.7)** | 0.25 |  |  |
| On tenofovir | **0.8 (0.4-1.4)** | 0.38 |  |  |
| Hypertension | **1.9 (1.0-3.6)** | 0.051 |  | ns |
| Triglycerides >2.6 mmol/L | **3.6 (1.8-7.2)** | **<0.001** | **2.4 (1.1-5.7)** | 0.036 |
| Non-HDL cholesterol >3.8 mmol/L | **1.5 (0.8-2.9)** | 0.20 |  | ns |
| Central obesity | **2.6 (1.3-5.2)** | 0.005 | **2.8 (1.2-5.7)** | 0.015 |
| Insulin resistance | **3.4 (1.8-6.5)** | **<0.0001** | **2.4 (1.1-5.1)** | 0.018 |
| Hs CRP >3.0 mg/L | **1.6 (0.8-3.2)** | 0.20 |  | ns |
| On statin treatment | **2.6 (1.4-4.9)** | 0.004 | **2.6 (1.2-5.5)** | 0.015 |
| Actual smoker | **0.9 (0.5-1.8)** | 0.81 |  |  |

Re: Consider adding sample sizes, n=570 and n=505 to your figure legends

**Response**: the figure legends have been changed to: “

***Figure 1.*** *Proportions of T1D, T2D and insulin resistance in the cross-sectional analysis at baseline (n=570).*

***Figure 2.*** *Proportions of T1D, T2D and insulin resistance in the prospectively followed cohort at follow up (n=505).*

Furthermore, the figure 2 has been corrected accordingly

Figure 1. Proportions of type 1 diabetes (T1D), type 2 diabetes (T2D) and insulin resistance in the cross-sectional analysis (n=570) at baseline

|  | Proportions of diabetes1, TD2 and insulin resistance in the trans-sectional study at baselin |
| --- | --- |
| T1D | 1.22 |
| T2D | 10.18 |
| Insulin resistance | 26.84 |
| Non-diabetes | 61.75 |

Figure 2. Proportions of type 2 diabetes (T2D) and insulin resistance in the prospectively followed cohort (n=505) at follow up.

|  | Proportions of diabetes1, TD2 and insulin resistance in the trans-sectional study at baselin |
| --- | --- |
| T2D | 8.5 |
| Insulin resistance | 37.5 |
| Non-diabetes | 54 |
