# Supplementary material for: Development of type 2 diabetes and insulin resistance in people with HIV infection: Prevalence, incidence and associated factors
Source: PLoS One. 2021 Jun 30;16(6):e0254079. doi: 10.1371/journal.pone.0254079 (PMC8244855; doi:10.1371/journal.pone.0254079)
Supplement: Attachment — Submitted filename: Response to the reviewers_R3.docx [file pone.0254079.s005.docx]

**Responses to the minor comments:**

**Reviewer #1: Minor Concerns:**

Re: Material and Methods
Inclusion criteria number 2 has been clarified to “Born before the 1st of January 2013.” However, this suggests that children as young as 8 years old could participate in the study. Please revise accordingly.

This is a typo, corrected to *born before the 1st of January 1963* in the revised manuscript

Re: Results:
The concerns regarding the incidence of insulin resistance at baseline in the prospective cohort were addressed but further clarity is needed. I would suggest the following changes addition to the second sentence of the paragraph “Another 36 patients, 10% (36/352) (M:n=19; F: n=17) developed insulin resistance (incidence of 1.0/100 patient-years).
In addition, the last sentence reads “In total, the occurrence of either T2D or insulin resistance was 46% (232 (153+79)/505) at follow up, Figure 2. I would suggest using ‘prevalence’ in place of ‘occurrence.’

We appreciate this comment and have now change the text accordingly in the revised manuscript. The development of insulin resistance during follow up was 7% (36 out of 505)

Re: Abstract
Please make the correction above to the abstract as well since the old data for insulin resistance is still reflected.

Thanks for notice this, the abstract is now changed.

Results: During follow up (3485 patient-years) 9% (43/505) developed T2D and 7% (34/505) insulin resistance.

This is now corrected in the Abstract (Results). “During follow up (3485 patient-years) 9% (43/505) developed T2D and 7% (36/505) insulin resistance”
